# Cytochrome P450-2E1 promotes fast food-mediated hepatic fibrosis

**DOI:** 10.1038/srep39764

**Published:** 2017-01-04

**Authors:** Mohamed A. Abdelmegeed, Youngshim Choi, Grzegorz Godlewski, Seung-Kwon Ha, Atrayee Banerjee, Sehwan Jang, Byoung-Joon Song

**Affiliations:** 1 Section of Molecular Pharmacology and Toxicology, Laboratory of Membrane Biochemistry and Biophysics, National Institute on Alcohol Abuse and Alcoholism, Bethesda, MD, 20892, USA; 2 Laboratory of Physiologic Studies, National Institute on Alcohol Abuse and Alcoholism, Bethesda, MD 20982, USA

## Abstract

Cytochrome P450-2E1 (CYP2E1) increases oxidative stress. High hepatic cholesterol causes non-alcoholic steatohepatitis (NASH) and fibrosis. Thus, we aimed to study the role of CYP2E1 in promoting liver fibrosis by high cholesterol-containing fast-food (FF). Male wild-type (WT) and *Cyp2e1*-null mice were fed standard chow or FF for 2, 12, and 24 weeks. Various parameters of liver fibrosis and potential mechanisms such as oxidative and endoplasmic reticulum (ER) stress, inflammation, and insulin resistance (IR) were studied. Indirect calorimetry was also used to determine metabolic parameters. Liver histology showed that only WT fed FF (WT-FF) developed NASH and fibrosis. Hepatic levels of fibrosis protein markers were significantly increased in WT-FF. The nitroxidative stress marker iNOS, but not CYP2E1, was significantly elevated only in FF-fed WT. Serum endotoxin, TLR-4 levels, and inflammatory markers were highest in WT-FF. FAS, PPAR-α, PPAR-γ, and CB1-R were markedly altered in WT-FF. Electron microscopy and immunoblot analyses showed significantly higher levels of ER stress in FF-fed WT. Indirect calorimetry showed that *Cyp2e1*-null-mice fed FF exhibited consistently higher total energy expenditure (TEE) than their corresponding WT. These results demonstrate that CYP2E1 is important in fast food-mediated liver fibrosis by promoting nitroxidative and ER stress, endotoxemia, inflammation, IR, and low TEE.

Nonalcoholic fatty liver disease (NAFLD) represents a hepatic metabolic syndrome^1^. NAFLD includes fatty liver (simple steatosis), steatohepatitis (NASH), fibrosis, and cirrhosis^2^. Obesity and chronic over-nutrition are risk factors for NAFLD^3^. The “two-hit” hypothesis for the progression of NASH^4^ postulates that the pathophysiology starts with steatosis (the primary hit), which primes the liver to oxidative stress (a secondary hit). Further studies revealed many other risk factors such as gut-derived endotoxins, pro-inflammatory cytokines, endoplasmic reticulum (ER) stress, and insulin resistance (IR) can also serve as the secondary hit^5^,^6^,^7^.

Dietary components play an important role in the development and progression of NAFLD/NASH^8^. The hepatic cholesterol content has gained much interest as an important dietary component that can cause NASH^9^. In addition, high dietary cholesterol can initiate the inflammation process, leading to NASH development and fibrosis^10^,^11^.

Cytochrome P450–2E1 (CYP2E1) metabolizes a variety of small molecule substrates including long-chain fatty acids^12^,^13^. Superoxide, the oxidative radical produced from CYP2E1-mediated metabolisms^12^,^13^, can serve as part of the second hit to advance the severity of NAFLD. CYP2E1 expression in the liver was increased in humans and animal models of NAFLD^12^,^14^,^15^. Furthermore, by using mice fed high-fat diet (HFD) for 10 weeks (wks), we and others reported an important role of CYP2E1 in the development of NASH^16^,^17^. However, it is unknown whether CYP2E1 is involved in the liver fibrosis caused by HFD containing cholesterol.

Therefore, the main aim of this study was to examine the hypothesis that CYP2E1 plays a role in the progression of NASH to liver fibrosis. The second aim was to elucidate the potential underlying mechanism(s) by which CYP2E1 promotes liver fibrosis. To achieve liver fibrosis, we used male wild-type (WT) and *Cyp2e1*-null mice fed a western fast-food (FF) [high-fat high-cholesterol diet, for 2, 12, and 24 wks]. Critical parameters under the expanded “two hit hypothesis” were examined as potential underlying mechanism(s) to explain the differential effects between WT and *Cyp2e1*-null mice fed FF, i.e., by examining hepatic steatosis “the first hit” and various “second hit” factors such as oxidative stress, inflammatory response, gut-derived serum endotoxin, ER stress, and IR, which can contribute to liver fibrosis^5^,^6^,^7^. Additionally, we studied the differential energy expenditure and ambulatory activities in both western FF-fed mouse strains by indirect calorimetry which may explain the different rates of body weight gain and hepatic steatosis in WT-FF and *Cyp2e1*-null-FF groups.

## Results

### Increased liver injury and body weight (B.W.) in WT-FF mice

**Figure 1:**
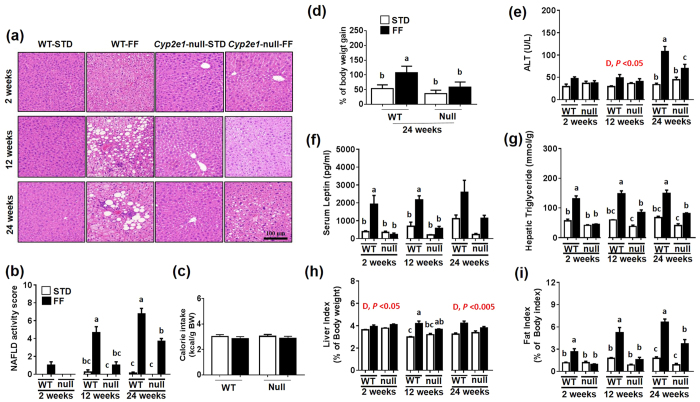
Increased histopathological hepatic injury and weight gain in WT-FF mice. (**a**) Representative H&E staining images for the livers of the indicated mouse groups fed for 2, 12, or 24 wks are presented. (**b**) NAFLD activity score, (**c**) caloric intake, (**d**) percentage of body weight gain at 24 wks, (**e**) serum ALT, (**f**) serum leptin, (**g**) hepatic TG, (**h**) liver index, and (**i**) fat index are shown (n = 4–5/group). D, diet; G, genotype. Columns without a common letter are significantly different from each other, *P* < 0.05.

Livers of all mice fed STD appeared visually (data not shown) and histologically normal (Fig. 1a), while livers from both WT-FF and *Cyp2e1*-null-FF exhibited a much paler color compared with those of STD controls. Histological analysis (Fig. 1a) revealed progressive accumulation of micro- and macro-vesicular intracellular lipid droplets and inflammatory foci in WT-FF and to a much lesser extent in *Cyp2e1*-null-FF mice than the corresponding STD groups. Histological scoring system for NAFLD^18^ revealed that only WT-FF group achieved an NAS of ≥5 at both 12 and 24 wks (Fig. 1b). A significant diet-genotype interaction for these two duration groups was observed and further statistical analysis showed that WT-FF was highest among all groups (Fig. 1b) despite the similar caloric intakes among all groups (Fig. 1c). There was a significant diet-genotype interaction for the percentage of BW gain and the BW of WT-FF group was significantly higher than all other groups (Fig. 1d). Serum ALT levels did not show significant difference among the groups at 2 wks (Fig. 1e). However, significant differences were noticed in both FF groups compared to their STD controls (diet effect) at 12 wks, while there was a significant diet-genotype interaction at 24 wks (Fig. 1e). Further analysis of 24 wks data by one-way ANOVA showed plasma ALT was highest in WT-FF, although ALT levels in *Cyp2e1*-null-FF were higher than the STD-controls (Fig. 1e). A significant diet-genotype interaction was observed for serum leptin at 2 and 24 wks, and WT-FF had significantly higher leptin levels than all other groups as validated by one-way ANOVA (Fig. 1f). At 24 wks, there were significant diet and gene effects on serum leptin levels since both FF groups exhibited higher levels than their corresponding controls and that the leptin level in WT-FF was ~3-folds higher than their corresponding *Cyp2e*1-null group (Fig. 1f). Analysis of TG levels showed a significant diet-genotype interaction for all examined durations (Fig. 1g). Moreover, TG levels were highest in WT-FF groups for 2, 12, and 24 wks, although TG levels in *Cyp2e1*-null-FF mice significantly higher than their STD controls at 12 and 24 wks (Fig. 1g). However, liver index was affected by the diet only at 12 and 24 wks since both FF mice groups had significantly higher ratios than mice fed STD (Fig. 1h). At 12 wks, there was a diet-genotype interaction for the liver index and further analysis showed that WT-FF mice exhibited significantly higher liver index than both WT and *Cyp2e1*-null-STD controls, but not observed when compared to the corresponding *Cyp2e1*-null-FF (Fig. 1h). In contrast, there was a significant diet-genotype interaction for 2, 12, and 24 wks when fat index was evaluated (Fig. 1i). Moreover, fat index was significantly higher in WT-FF than all other groups for all feeding periods although it was significantly higher in *Cyp2e1*-null-FF mice than their corresponding STD-controls at 24 wks (Fig. 1i).

### WT-FF mice develop hepatic fibrosis

**Figure 2:**
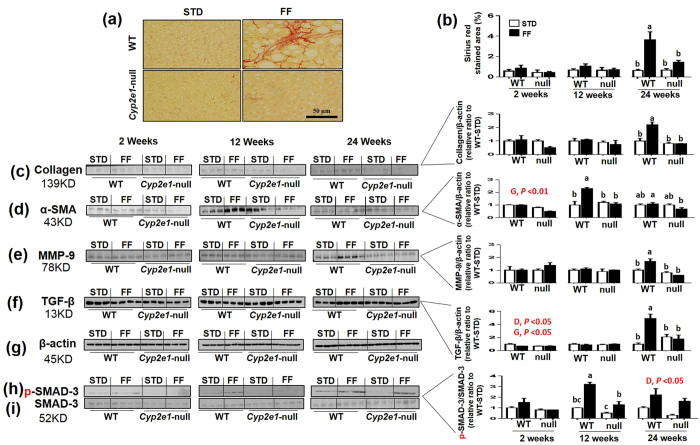
Development of hepatic fibrosis in WT-FF mice. (**a**,**b**) Representative Sirius red staining for collagen in hepatic sections (*n* = 4–5/group) at 24 wks of feeding and percentage of fibrotic areas, respectively, following 2–24 wks of feeding. Immunoreactive levels and densitometric analyses for (**c**) collagen, (**d**) α-SMA, (**e**) MMP-9, (**f**) TGF-β and (**g**) the loading control β-actin are shown. (**h**) Images and densitometric analysis of P-SMAD-3 and (**i**) the loading control SMAD-3. Values of WT-STD were set at 1 as controls (*n* = 3–4/group). D, diet; G, genotype. Columns without a common letter are significantly different from each other, *P* < 0.05.

Hepatic fibrosis, as examined by Sirius red staining, revealed a significant diet-genotype interaction at 24 wks (Fig. 2a,b). One-way ANOVA analysis suggested that the percentage of Sirius red-stained areas was significantly higher in WT-FF than all other groups (Fig. 2a,b). Further, the accumulation of collagen fiber and development of hepatic fibrosis in WT-FF mice were confirmed by immunoblot analysis using the specific anti-collagen 1A1 antibody (Fig. 2c) and by RT-PCR analyses for 24-wks (Supplementary (S-) Fig. 1a), which exhibited a similar pattern of Sirius red staining at 24 wks. The level of α-SMA was significantly lower in *Cyp2e*1-null mice than WT at 2 wks (i.e. gene effect) (Fig. 2d). A significant diet-genotype interaction was observed at 12 and 24 wks (Fig. 2d). However, the highest levels were reported in WT-FF at 12 wks, while at 24 wks, the significant diet-genotype interaction was observed due to the lower levels of α-SMA expression in *Cyp2e1-*null-FF than their corresponding WT-FF (Fig. 2d). Immunoblot analyses with specific antibodies against MMP-9 and TGF-β exhibited similar patterns of both proteins with statistical significance (significant diet-genotype interaction followed by significant differences by one-way ANOVA) similar to that of collagen at 24 wks (Fig. 2e,f), that was also confirmed by RT-PCR (S-Fig. 1B). However, there was a significant effect of diet and gene on the expression of TGF-β as its protein levels in Cyp2e1-FF were lower than in the corresponding STD control, although its contents in WT-FF were significantly elevated than those of WT-STD after 24 wks of feeding (Fig. 2f). Normalized values of each protein against β-actin (Fig. 2g), used as a loading control, are shown, as indicated. P-SMAD-3, a critical downstream signaling molecule of TGF-β, exhibited a diet-genotype interaction at 12 wks, but not at 2 wks. Further analysis revealed significantly higher levels of P-SMAD-3 in WT-FF than any other groups (Fig. 2h). However, *Cyp2e1*-null-FF exhibited significantly higher levels of p-SMAD3 than their STD-controls (Fig. 2h). AT 24 wks, both FF mouse groups exhibited significant higher levels than their corresponding control groups (i.e. diet effect) (Fig. 2h). SMAD-3 (Fig. 2i) was used as a loading control for P-SMAD-3 and the pSMAD-3/SMAD-3 values are shown.

After observing significantly higher hepatic steatosis (1^st^ hit) and the end point hepatic fibrosis in WT-FF, we therefore evaluated potential 2^nd^ hit factors such as oxidative stress, endotoxemia, hepatic inflammation, ER stress, and insulin resistance.

### Increased levels of iNOS, but not CYP2E1, in WT-FF mice

**Figure 3:**
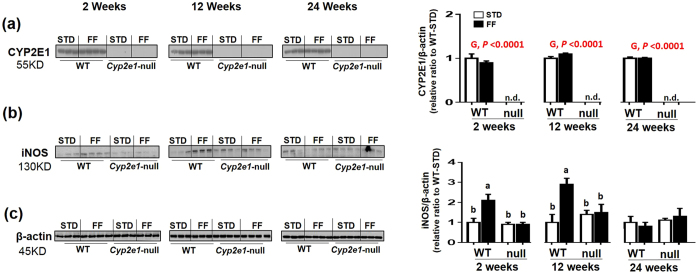
Increased hepatic iNOS protein levels, but not CYP2E1 in WT-FF mice. (**a**,**b**) Immunoblot images and densitometric analyses for CYP2E1 and iNOS, respectively, normalized to β-actin (**c**). Values for WT-STD were set at 1 as controls. G, genotype. Columns without a common letter are significantly different from each other, *P* < 0.05.

As expected, there was a significant difference in CYP2E1 protein between WT and *Cyp2e1*-null mice (gene effect) for all groups (Fig. 3a). Interestingly, CYP2E1 protein levels were not increased in WT-FF mice (Fig. 3a). In addition, CYP2E1 activities in the WT-FF mice were not significantly increased in this model (data not shown but shown to the reviewers). However, significant diet-genotype interactions in the iNOS protein levels were observed at 2 and 12 wks, but not 24 wks (Fig. 3b). One-way ANOVA revealed significantly higher levels of iNOS protein levels in WT-FF than all other groups at 2 and 12 wks (Fig. 3b). Normalized values of both proteins against β-actin (Fig. 3c), used as a loading control, are presented, as indicated.

### Development of endotoxemia and hepatic inflammation in WT-FF mice

**Figure 4:**
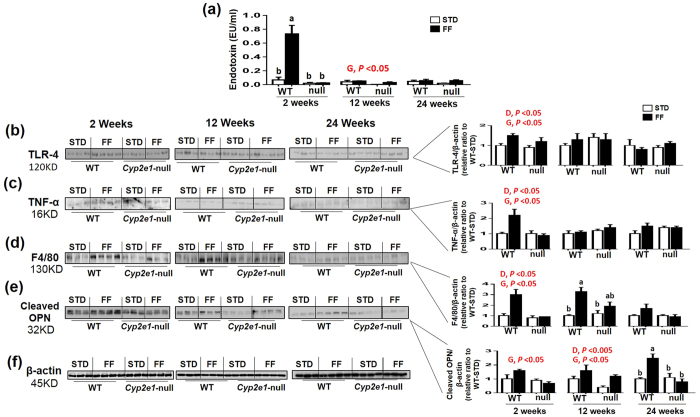
Increased serum endotoxin and hepatic markers of inflammation in WT-FF. (**a**) Tabulated serum endotoxin levels. Immunoblot images and densitometric analyses for (**b**) TLR-4, (**c**) TNF-α, (**d**) F4/80, and (**e**) cleaved osteopontin, normalized to β-actin (**f**). Values for WT-STD were set at 1 as controls (*n* = 3–4/group). D, diet; G, genotype. Columns without a common letter are significantly different from each other, *P* < 0.05.

There was a significant diet-genotype interaction for serum endotoxin levels at 2 wks, while the levels of endotoxin in WT groups were significantly higher than the corresponding *Cyp2e1*-null groups at 12 wks (i.e. gene effect), but not at 24 wks (Fig. 4a). One-way ANOVA showed that serum endotoxin levels were highest in WT-FF at 2 wks (Fig. 4a). Immunoblot analysis of TLR4, TNF-α, and F4/80 revealed that there were significant effects of both the gene and diet only at early time point (2 wks) (Fig. 4b–d), while there was a significant effect of the diet on F4/80 at 12 wks (Fig. 4d). The levels of the cleaved (active form) OPN showed that there was a significant gene effect at 2 wks, a significant diet and gene effect at 12 wks, and diet-genotype interaction at 24 wks (Fig. 4e). Moreover, cleaved OPN level was highest in WT-FF among all groups at 24 wks (Fig. 4e). Normalized values for each protein against β-actin (Fig. 4f), used as a loading control, are presented.

### Changes in the levels of hepatic proteins involved in lipid homeostasis

**Figure 5:**
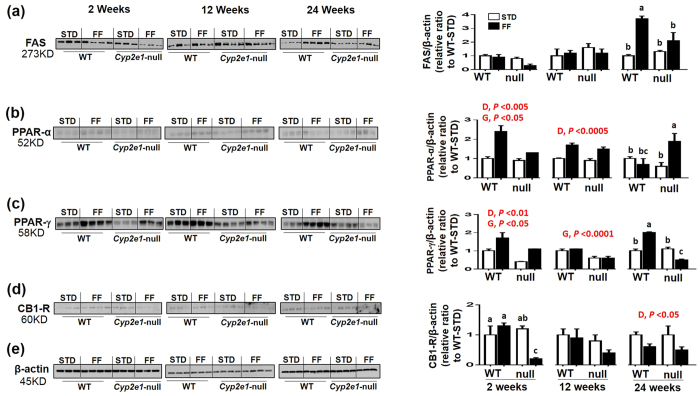
Alteration of hepatic proteins involved in lipid homeostasis in FF-fed mice. Immunoblot images and densitometric analyses for (**a**) FAS, (**b**) PPAR-α, (**c**) PPAR-γ, and (**d**) CB1-R, normalized to β-actin (**e**) are shown. Values for WT-STD were set at 1 as controls (*n* = 3–4/group). D, diet; G, genotype. Columns without a common letter are significantly different from each other, *P* < 0.05.

We found that FAS exhibited a significant diet-genotype interaction only at 24 wks (Fig. 5a). One-way ANOVA suggested that WT-FF had the highest levels of fatty acid synthase (FAS) (Fig. 5a). Both PPAR-α and PPAR-γ exhibited significant gene and diet effects at 2 wks since both protein levels were higher in WT-FF group than other groups (Fig. 5b,c, respectively). However, at 12 wks, PPAR-α expression was affected by diet as its levels were significantly higher in both FF-fed mouse groups (Fig. 5b). In contrast, PPAR-γ expression was affected by gene since its expression was significantly higher in both WT groups than *Cyp2e1*-null groups (Fig. 5c). There was a significant diet-genotype interaction at 24 wks that affected both PPAR-α and PPAR-γ (Fig. 5b,c, respectively). Further one-way ANOVA showed that PPAR-α and PPAR-γ protein levels were highest in *Cyp2e1-*null-FF and WT-FF, respectively, among all groups (Fig. 5b and c). A significant diet-genotype interaction was found for CB1-R protein expression at 2 wks, and one-way ANOVA revealed that CB1-R levels were significantly lower in *Cyp2e1*-null-FF than all other groups (Fig. 5d). At 12 wks, there was no significant difference on CB1-R expression although its level was lowest in *Cyp2e1*-null-FF. In contrast, there was a significant diet effect at 24 wks since CB1-R levels were lower in both FF-fed mouse groups than their corresponding STD-controls (Fig. 5d). Normalized values of each protein against β-actin (Fig. 5e), used as a loading control, are presented.

### Development of ER stress in WT-FF

**Figure 6:**
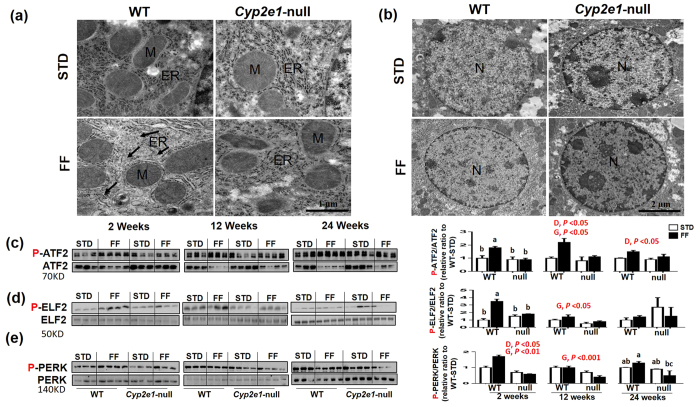
Increased levels of ER stress proteins in WT-FF. Transmission EM analysis demonstrates (**a**) regularly organized ER (ER) (STD) and irregularly arranged and/or disrupted ER in FF (FF), which are more prominent in WT-FF (*n* = 3–4/group). (**b**) Nuclei are shown for all groups. Immunoblot images and densitometric analyses for (**c**) P-ATF-2, (F, upper) P-ELF-2, and (G, upper) P-PERK, normalized to their corresponding protein as indicated. Values for WT-STD were set at 1 as controls (*n* = 3–4/group). D, diet; G, genotype. Columns without a common letter are significantly different from each other, *P* < 0.05.

Transmission EM analysis of the specimens obtained after 24 wks of feeding demonstrated regularly organized ER in close association with mitochondria in both STD-fed control groups (Fig. 6a,b and Sup-Fig. 2). The levels of irregularly-arranged and disrupted ER in FF-fed WT were greater than those of *Cyp2e1*-null counterparts while there were no apparent marked differences in the nucleus or mitochondria between the groups, other than slight changes in the shape and size of the mitochondria in FF groups (Fig. 6a,b and Sup-Fig. 2). At 2 wks, there was a significant diet-genotype interaction for both ATF-2 and P-ELF-2 and significant diet and gene effects for P-PERK (Fig. 6c–e respectively). Further analysis by one-way ANOVA for P-ATF-2 and P-ELF-2 showed that the amounts of both proteins were highest in WT-FF among all other groups (Fig. 6c,d respectively). At 12 wks, there was a significant diet and gene effect on the expression of P-ATF-2 as the levels of this protein in WT-FF were ~2- to 2.7-fold higher than all other groups (Fig. 6c). However, there was only a gene effect at 12 wks for both P-ELF-2 and P-PERK since their levels were lower in *Cyp2e1*-null groups than in WT (Fig. 6d,e). At 24 wks, there was a significant diet effect on P-ATF-2 expression since the levels of this protein were higher in both mouse groups fed FF (Fig. 6c). The levels of P-ELF-2 at 24 wks were not significantly changed (Fig. 6d). However, there was a significant diet-genotype interaction for P-PERK (Fig. 6e). One-way ANOVA showed that P-PERK levels were highest in WT-FF (Fig. 6e). Normalized values of phosphorylated active proteins against their non-phosphorylated ATF-2, ELF-2, and PERK, used as loading controls (Fig. 6c–e, lower panels), are shown.

### WT-FF mice have IR and reduced GT

**Figure 7:**
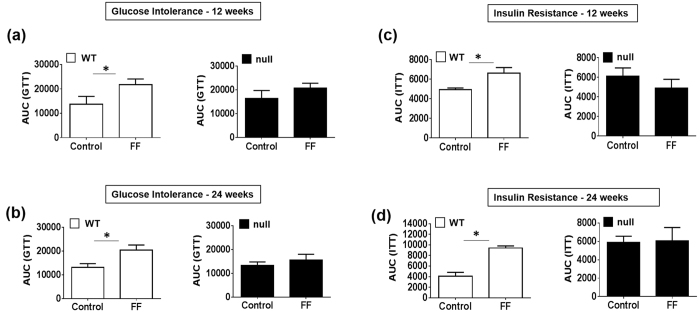
Impaired GT and increased IR in WT-FF. (**a**,**b**) Tail blood was collected following glucose injection (i.p., 2 g/kg) or (C and D) insulin injection (i.p., 0.75 U/kg; Eli Lilly) at indicated times, and total AUC values were tabulated (*n* = 4/group). *Significantly different from control, *P* < 0.05.

Our results revealed that WT-FF mice developed significantly impaired GT (Fig. 7a,b and S-Fig. 3A,B) and systemic IR (Fig. 7c,d and S-Fig. 3C,D) compared to the WT-STD groups at 12 and 24 wks, respectively. In contrast, no significant differences were observed in GT (Fig. 7a,b and S-Fig. 3A,B) or insulin sensitivity (Fig. 7a,b and S-Fig. 3C,D) in *Cyp2e1-*null-FF mice compared to the corresponding STD-exposed groups.

### *Cyp2e1*-null mice have increased total energy expenditure

To shed a light on potential metabolic reasons of increased body weight gain and hepatic steatosis in WT-FF than *Cyp2e1*-null-FF, we performed indirect calorimetry study on both mouse groups fed FF. Our results revealed significant increases in the total energy expenditure (TEE) in *Cyp2e1*-null-FF mice compared to their WT counterparts at 2 and 12 wks and the trend was similar (*p* = 0.074) at 24 wks (S-Figs 4–6C). This was accompanied with significant concomitant increases in oxygen consumption and carbon dioxide production in *Cyp2e1*-null-FF mice compared to their WT-FF for all time groups (S-Figs 4–6A,B, respectively). Rates of fat oxidation (FO) was significantly higher in *Cyp2e1*-null-FF at 12 wks and the similar trends were observed at 2 and 24 wks (*p* = 0.067 and 0.076, respectively) (S-Figs 4–6D), consistent with the elevated levels of PPAR-α in *Cyp2e1*-null-FF (Fig. 5). Importantly, the ambulatory activity (AA) exhibited no difference at 2 wks (S-Fig. 4E), while it was significantly higher in *Cyp2e1*-null-FF at 12 wks (S-Fig. 5E). A similar trend was observed at 24 wks, although the difference was not significant (S-Fig. 6E).

## Discussion

The aim of this study was to investigate the role of CYP2E1 in fast food-induced development of NASH and fibrosis by comparing histological and biochemical parameters in WT and *Cyp2e1*-null mice. Long-term FF feeding in WT, but not *Cyp2e1*-null mice, produced typical features of NASH indicated by elevated steatosis (1^st^ hit), nitroxidative stress, inflammation, liver injury and fibrosis. This was evidenced by histological analysis and increased levels of serum ALT (Figs 1 and 2). NASH phenotype was associated with liver fibrosis, obesity, hyperleptinemia, increased oxidative and ER stress, inflammation, and increased IR and impaired GT (multiple 2^nd^ hits) in WT-fed FF compared to the corresponding *Cyp2e1*-null mice. These effects were not due to differences in caloric intake (Fig. 1c), suggesting that other biochemical and metabolic responses may be the underlying reasons for the differential effects of FF on WT and *Cyp2e1*-null mice. Cholesterol might accelerate NASH development since dietary cholesterol is rapidly transferred to liver following absorption^19^, and induces arterial plaques formation that increases the levels of inflammatory cytokines^20^. Indeed, the addition of 0.2% cholesterol to HFD induced NASH in mice when supplemented with sucrose in the drinking water^21^.

The genetic deletion and/or inhibition of CYP2E1 was shown to prevent/reduce hepatic steatosis in AFLD and NAFLD models^16^,^17^,^22^,^23^,^24^, which was mainly attributed to the development of nitroxidative stress and in some cases the alteration of proteins involved in lipid homeostasis such as PPAR-α^22^,^23^,^24^. Interestingly, levels of proteins involved in fatty acid oxidation, and have anti-inflammatory functions, as PPAR-α and PPAR-γ^15^,^25^, were increased more prominently in WT-FF groups fed at early points (2 wks), which may be an adaptive cellular response to the increased intake of FF (consisting of HFD and cholesterol). However, PPAR-α levels decreased over time in WT-FF, while it increased in *Cyp2e1*-null-FF group (12 and 24 wks). The decreased PPAR-α levels in WT-FF over time may have been compensated partially with the increased levels of PPAR-γ. However, this increase might not be sufficient enough to accommodate the increased high fat pool in the WT, since FAS was also shown to increase particularly at late time points. Together, this might overwhelm the compensatory cellular accommodation in WT-FF. In addition, hepatic CB1 receptor, critical for the development of HFD-induced steatosis, dyslipidemia, and insulin and leptin resistance in mice as demonstrated by using WT and liver specific *CB1*-null mice fed HFD^26^, was found to be consistently lower in *Cyp2e1*-null-FF, suggesting a potential interplay between CB1 and CYP2E1 in the regulation of lipid homeostasis in response to diet rich in fat and cholesterol. In addition, we evaluated potential roles of differential metabolism–related activities in both mouse strains fed FF using indirect calorimetry analyses to explain the differences in both body weight gain and hepatic steatosis. Interestingly, *Cyp2e1*-null-FF mice exhibited overall higher TEE, increased ambulatory activity, and fatty acid oxidation efficiency than their WT-FF counterparts (S-Figs 4–6), probably explaining the underlying reasons for the leaner *Cyp2e1*-null-FF mice with lower body weight gain and hepatic steatosis. Further studies are required to fully characterize the underlying mechanisms for the differential metabolic responses in WT and *Cyp2e1*-null groups.

The development of fibrosis in WT-FF was confirmed both histologically and biochemically (Fig. 2), as known markers and mediators of liver fibrosis were significantly upregulated in WT-FF mice such as collagen-1A1, α-SMA, TGF-β, and MMP-9^27^. For instance, MMP-9 was thought to play an important role in the activation of inflammatory cytokines and quiescent hepatic stellate cells through its proteolytic activity^28^,^29^. It is noteworthy to mention that the time of upregulation of the evaluated profibrogenic proteins in this study was not always identical. This suggests that the process of fibrogenesis is a temporal and dynamic process.

Surprisingly, we did not observe significant changes in the levels of CYP2E1, as a source of increased oxidative stress, in WT-FF at any time point (Fig. 3), possibly due to the high carbohydrate content (~44% energy derived from carbohydrate) in the AIN-76A Western FF we used, as reported earlier^30^,^31^. This result is in contrast to many studies which reported increased levels of CYP2E1 expression in human specimens and animal models of NAFLD^12^,^14^,^15^,^16^,^17^. This led us to assume that the presence of CYP2E1 even at basal levels might be adequate enough to induce the hepatic damaging effect through a permissive role to either upregulate other proteins or mediate their damaging effects, as reported^32^. Recently, following the use of high cholesterol diet (HCD), iNOS was upregulated, and was suggested as an important mediator of liver fibrosis since it was developed in WT mice, but not in *iNOS*-null fed HCD mice^11^. In agreement, we found that iNOS levels were higher in WT-FF, but not in *Cyp2e1*-null-FF (Fig. 3). In addition, a recent seminal study showed that hybrid inhibitor of peripheral CB1-R and iNOS mitigates liver fibrosis in many murine models of fibrosis^33^, in which many models of fibrosis were used. These results support our suggestion of the important role of CB-1-R and iNOS in the protection of *Cyp2e1*-null mice from FF-induced liver fibrosis. Collectively, these results suggest that *Cyp2e1* might play a permissive role, through increased ROS production albeit small amounts, for iNOS upregulation to mediate its hepatic damaging effects. Whether CYP2E1 directly and/or indirectly, via its permissive action through other factors such as iNOS, promotes the development of liver fibrosis and whether CYP2E1 plays a role in regulating iNOS and CB-1-R warrant further investigation. The lack of upregulated CYP2E1 in our current model despite its clear involvement in the development of hepatic fibrosis may echo some of the discrepancies found in the literature about the role of CYP2E1 in mediating NASH in humans. Our results suggest that the inconsistently upregulated CYP2E1 or the lack of significant correlation between CYP2E1 levels and NASH or the CYP2E1 variant allele (CYP2E1*5) and NASH as discussed^34^, should not automatically exclude the involvement of CYP2E1, based on the clearly different levels of hepatic fibrosis between the WT and the corresponding *Cyp2e1*-null mice fed the same AIN-76A Western FF.

Obesity, HFD, and diet rich in cholesterol can produce inflammatory cytokines and increase hepatic inflammation in steatotic livers^5^,^11^. Furthermore, CYP2E1 sensitizes hepatocytes to lipopolysaccharides (LPS), TNF-α, and oxidative injury in mice treated with pyrazole and LPS^35^. We found significantly higher levels of serum endotoxin and its receptor TLR-4, inflammatory cytokines TNF-α and activated OPN, and macrophage marker F4/80 in WT-FF particularly at early time points (2 or 12 wks) (Figs 1 and 4)^5^. Studies suggested a critical role of LPS/TLR-4 signaling axis in the inflammatory pathways associated with NASH^36^, and the Inhibition of LPS-TLR4 signaling with antibiotics attenuated liver fibrosis development in rat NASH model^36^. In addition, it was shown that OPN levels are upregulated or activated (cleaved) in tissue and blood of patients with chronic liver disease, and OPN activates liver progenitors and hepatic stellate cells, which are critically important in liver fibrosis and formation of scar tissues^37^. OPN upregulation was reported to be involved in fibrosis in murine models^38^,^39^. One of the proposed mechanisms of OPN-induced hepatic fibrosis was via the enhancement of leptin-mediated fibrogenesis^39^. Consistently, we also observed significantly higher serum leptin levels in WT-FF at 2 and 12 weeks (Fig. 1f). This is also in agreement with another study where diet-induced obesity plus bromodichloromethane, a substrate for CYP2E1, promoted NASH and increased collagen levels partly due to the presence of CYP2E1 and leptin^40^. Together, it is intriguing to assume that OPN-mediated leptin effects on liver fibrosis^39^ requires the expression of CYP2E1, although this warrants further investigation.

Under pathological conditions including NASH, the ER function in the protein-folding, repairing, and/or trafficking machinery is significantly suppressed, leading to the development of ER stress although there is an increased demand of protein synthesis and folding/repair^41^. This might result in the activation of stress signaling pathways, which can stimulate ER stress proteins, such as PERK, ELF-2, and ATF-2. Our results showed more dramatic disarray in ER in WT-FF and the upregulation of these three proteins (Fig. 6). When combined with oxidative stress, inflammation, and insulin resistance, hepatic ER stress seems to play an important role in regulating the composition and size of lipid droplets as well as lipid synthesis, including cholesterol metabolism^41^,^42^. In agreement, *in vivo* and *in vitro* studies revealed that berberine prevents progression from hepatic steatosis to NASH and fibrosis by reducing ER stress^43^. Thus, elevated ER stress might also be part of the second hit that worsens NASH condition.

**Figure 8:**
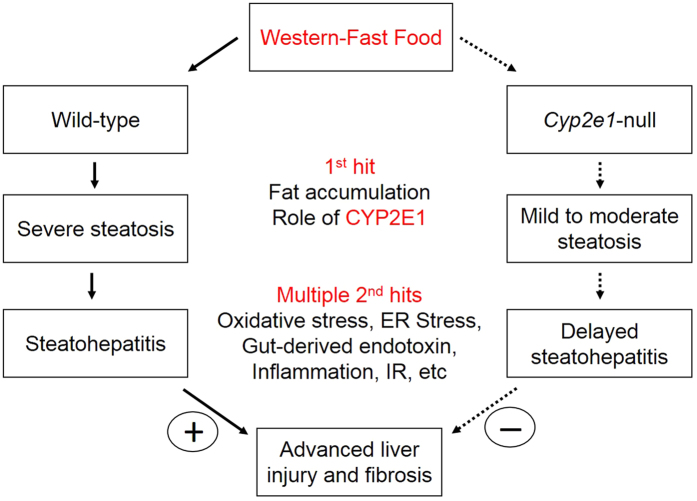
Schematic diagram for the role of CYP2E1 in western FF-mediated liver fibrosis. The first and second hits for the development of NASH and fibrosis by western fast food are presented. CYP2E1 seems to play a critical role in elevating the first hit, fat accumulation, and the development of advanced stages of NASH with liver fibrosis through multiple second hits such as increased nitroxidative stress, ER stress, gut derived endotoxin levels, inflammation, and insulin resistance (IR). The dotted arrows refer to delayed or little development.

Obesity, NAFLD, NASH, and HFD contribute to the high incidence of IR, which may accelerate the progression of NASH due to increased levels of oxidative stress, inflammation, and ER stress^5^,^16^,^17^. Furthermore, it was shown that over-expression of hepatic CYP2E1 *in vitro* and *in vivo* promoted the development of hepatic IR^44^,^45^, leading to NASH and fibrosis, as similar to those in age-dependent fibrosis^46^. These results support the critical role of CYP2E1 in hepatic fibrosis in two different models namely caused by western FF and aging. In agreement, only WT-FF mice developed increased IR and impaired GT at 12 and 24 weeks (Figs 7 and 8 and S-Fig. 3), supporting the notion that the absence or blocking of CYP2E1 may be beneficiary for not only preventing increased IR and impaired GT but also preventing advanced liver disease such as NASH and fibrosis.

It is important to recognize that various second hits do not necessarily take place simultaneously and that fibrosis can develop after these hits were combined. The development of fibrosis is a slow, chronic process and the effects of multiple second hits can be cumulative, leading to sustained injury over a long period of time. It is also possible that one of the second hits may start before the other factors and might even activate them. It is also important to emphasize that the sustained elevation of a certain insult is not always needed to play a causal role in liver fibrosis as elevated endotoxin was only observed at 2 weeks. It is still important, however, to characterize the most prominent factor in the initiation and/or the activation of many of the second hits. CYP2E1 turned to be the most critical factor for stimulating many of the second hits examined in this study and may be a good therapeutic target, although additional research need to be undertaken to characterize the exact mechanism(s) by which CYP2E1 mediates its multiple effects.

NASH pathophysiology is a complex, time-dependent event and would result from the concerted effects of many initial causes, which subsequently stimulate various harmful pathways, leading to the final injury (fibrosis/cirrhosis). We evaluated the several key parameters of the expanded two-hit hypothesis at multiple time points for up to 24 weeks in a physiologically relevant model to study the role of CYP2E1 in fast food-mediated liver injury, mimicking human NASH patients. The evaluation of several parameters at multiple time points up to 24 weeks without the use of chemicals such as carbon-tetrachloride and avoiding the nutritional deficiency associated with a methionine/choline-deficient diet^39^, that does not reflect the conditions of human NASH patients, allowed us to demonstrate the sequential biochemical and pathological changes in the presence or absence of CYP2E1. Our results were also supported by indirect colorimetric measurements for the first time that *Cyp2e*1-null mice-FF exhibited an overall higher TEE, FO and AA, all of which can facilitate the development of hepatic steatosis. The suggestion of the involvement of the expanded two-hit hypothesis in mediating CYP2E1-induced fibrosis are interesting and can help in directing the efforts towards which of these factor(s) are the most critical. Of particular interest, and as suggested by indirect calorimetric measurements, that show for the first time that *Cyp2e*1-null mice-FF higher TEE, FO and AA might indicate that the consumption of unhealthy diet needs other supportive factors to induce its deleterious effects such as lack of mobility, as shown with WT-FF mice.

In summary, CYP2E1, which appears to be involved in increased body weight gain and hepatic fat accumulation in WT-fed FF, possibly partly due to lower fatty acid oxidation, impaired TEE and ambulatory activity, plays an important role in the development and advancement of NAFLD to NASH and fibrosis Our current results demonstrate that CYP2E1, either directly or indirectly via permissive action, elevates the levels of oxidative stress, ER stress, gut-derived endotoxemia, inflammation, and IR, all of which can negatively affect the vital functions of hepatocytes, contributing to the development of NASH and liver fibrosis (Fig. 8).

## Materials and Methods

### Materials

All chemicals were highest grades and from Sigma Chemical (St. Louis, MO, USA), unless indicated otherwise. The secondary antibodies and the primary antibodies specific to collagen and α-smooth muscle actin (α-SMA) were from Santa Cruz Biotechnology (Santa Cruz, CA, USA). Specific antibodies to CYP2E1, iNOS, osteopontin (OPN), transforming-growth factor-β (TGF-β), matrix metalloproteinase-9 (MMP-9), F4/80, tumor necrosis factor-alpha (TNF-α), peroxisome proliferator-activated receptor (PPAR)-α or -γ antibodies were from Abcam Inc. (Cambridge, MA, USA). Anti-cannabinoid 1 receptor (CB1-R) antibody was from Cayman Chemical (Ann Arbor, Michigan, USA). All other antibodies were from Cell Signaling Technology Inc. (Boston, MA, USA).

### Animal treatment and histopathology and electron microscopy analysis

All animal procedures were approved by the Institutional Animal Care and Use Committee of NIAAA, NIH, and the experiments were carried out in accordance with the accepted guidelines.

Age-matched (4~5 wks) male WT and *Cyp2e*1-null mice on 129/Svj background^47^ were fed either standard rodent chow (STD) or AIN-76A Western Diet [fast food (FF)] (40% energy-derived calories from fat with 0.2% w/w cholesterol) (TestDiet, St. Louis, MO, USA) *ad libitum* for 2, 12, or 24 wks. Mice (*n* = 4~5 per group) were randomly assigned to four groups per each time point: (1) WT fed STD (WT-STD); (2) WT-FF; (3) *Cyp2e1*-null fed STD (null-STD); and (4) null-FF. The diet composition is shown in Supplementary Table 1. The mice were housed at 22 °C with a 12 h light/dark cycle and given free access to diet and water. The weights of the mice were monitored before the start of the STD/FF regimen, once every wk, and at the time of euthanasia. After 2, 12, or 24 wks of feeding, the livers tissues were excised from mice fasted overnight (16 h). Liver index (liver weight/B.W.) and fat index (fat weight/B.W.) were calculated. Tissue preparation for histological examination and storage for future characterizations were performed, as described^15^. The histological evaluation and fibrosis scoring were performed blindly by an experienced pathologist. The NAFLD histological scoring system (NAS)^18^ was used to evaluate the liver samples. NASH was defined when NAS was ≥5. To evaluate hepatic fibrosis, formalin-fixed liver tissue sections were stained with Sirius red and the image was captured by Olympus BX-51 microscope equipped with Digital Camera DP-70 (Olympus, Japan). Fibrotic and total areas were determined and the percentage of fibrotic area was calculated using Image J software (National Institutes of Health, Bethesda, MD, USA) from various image fields per slide. Slices of liver tissues were fixed in modified Karnovsky’s fixative (2.5% glutaraldehyde and 2% paraformaldehyde in 0.15 M sodium cacodylate), and were sent to the Department of Pathology, Texas A&M University, USA for further processing and evaluation by transmission electron microscopy (EM).

### Data evaluation

Data represent results from at least two separate measurements, unless otherwise stated. Each data point represents the mean ± SEM, *n* = 4–5/group. Statistical analyses were performed using Prism6 software. For multiple groups, two-way ANOVA was performed to assess the differences for the diet (D) or the genotype (G) unless otherwise indicated, as previously described^15^,^46^. The effect of diet (D) or gene (G) is considered significant when P < 0.05 following two-way ANOVA. When two-way ANOVA revealed the presence of genotype–diet interaction, we performed one-way ANOVA followed by *post hoc* Tukey’s test at a significance level of 0.05 to determine the differences between the four groups, as previously described^15^,^47^. Any column (group), that does not share a common alphabetical letter above with other column(s), represent significantly different from that column (group) following the one-way ANOVA followed by *post hoc* Tukey’s test.

Other Methods are described in details in the Supplementary Materials and Methods.

## Additional Information

**How to cite this article**: Abdelmegeed, M. A. *et al*. Cytochrome P450-2E1 promotes fast food-mediated hepatic fibrosis. *Sci. Rep.* **7**, 39764; doi: 10.1038/srep39764 (2017).

**Publisher's note:** Springer Nature remains neutral with regard to jurisdictional claims in published maps and institutional affiliations.

## Supplementary Material

Supplementary Information
