# Corrigendum: Cytochrome P450-2E1 promotes fast food-mediated hepatic fibrosis

**DOI:** 10.1038/srep42566

**Published:** 2017-02-17

**Authors:** Mohamed A. Abdelmegeed, Youngshim Choi, Grzegorz Godlewski, Seung-Kwon Ha, Atrayee Banerjee, Sehwan Jang, Byoung-Joon Song

Scientific Reports 7: Article number: 3976410.1038/srep39764; published online: 01 04 2017; updated: 02 17 2017

In the Supplementary Information file originally published with this Article, Supplementary Table 1 was omitted. This error has now been corrected in the Supplementary Information that now accompanies the Article.

