# Supplementary material for: Cytochrome P450-2E1 promotes fast food-mediated hepatic fibrosis
Source: Sci Rep. 2017 Jan 4;7:39764. doi: 10.1038/srep39764 (PMC5209674; doi:10.1038/srep39764)
Supplement: Supplementary Information [file srep39764-s1.pdf]

SUPPLEMENTARY INFORMATION

Cytochrome P450-2E1 promotes fast food-mediated hepatic fibrosis

Mohamed A. Abdelmegeed[a*], Youngshim Choi[a], Grzegorz Godlewski[b], Seung-Kwon Ha[a], Atrayee Banerjee[a], Sehwan Jang[a], and Byoung-Joon Song[a*]

[a]Section of Molecular Pharmacology and Toxicology, Laboratory of Membrane Biochemistry and Biophysics, National Institute on Alcohol Abuse and Alcoholism, Bethesda, MD, 20892, USA, [b]Laboratory of Physiological Studies, National Institute on Alcohol Abuse and Alcoholism, Bethesda, MD 20982, USA.

Supplementary Materials and Methods

Blood chemistry

Serum alanine aminotransferase (ALT) was quantified by a clinical chemistry analyzer (IDEXX Vet Test, IDEXX Laboratories, Westbrook, ME, USA). Serum leptin was evaluated by Mouse Leptin ELISA Kit, Abcam Inc., Cambridge, MA, USA). Endotoxin levels were measured using the commercial kit from Lonza (Walkersville, MD, USA). Measurements for leptin and endotoxin were by following the manufacturers' instructions.

Tissue extraction, immunoblot analyses and measurements of hepatic TG content and CYP2E1 activity

Liver homogenates were prepared in ice cold extraction buffer (50 mM Tris–Cl, pH 7.5, 1 mM EDTA, and 1% CHAPS), as described[1]. Equal amounts of liver homogenates were resolved on 12% SDS–PAGE gels and immunoblot procedures were performed, as previously described[2]. Image detection was performed using a SuperSignal West Pico Kit

(Pierce) according to the manufacturer's instructions. β-Actin was used as a loading control, unless otherwise indicated. Values described on top of each immunoblot represent densitometric measurements normalized to the loading control, and the values of WT-STD were set at 1 as controls for all other groups at different time points. For hepatic TG measurement, liver tissues (50 mg wet weight) from individual mice of different groups were homogenized in 5% Triton X-100 solution and heated in 80–100°C water bath for 2–5 min to solubilize TG. The samples were then centrifuged at 10,000 × *g* for 10 min, and the resulting supernatants were used to determine the TG levels using the manufacturer's instruction provided with the EnzyChromTM TG Assay Kit (BioAssay Systems, Hayward, CA, USA). CYP2E1 activities were measured by quantifying the oxidation rate of *p*-nitrophenol (PNP) to *p*-nitrocatechol, as previously described[3].

IR and glucose tolerance (GT) tests

After 11 and 23 wks of feeding, IR and GT tests were performed in mice fasted for 6 h and subsequently injected with insulin (0.75 U/kg; Eli Lilly) and in mice fasted overnight and injected with glucose (2 g/kg), respectively. GT tests were performed 3 days after the IR tests. Tail blood was collected at 0, 30, 60, 90, and 120 min after intraperitoneal injection of insulin (IR) or glucose (GT). Blood glucose levels were determined using the Elite glucometer (Bayer), as detailed[2].

Indirect calorimetry and ambulatory activity.

Measurements for both WT and *Cyp2e1*-null mice fed FF were conducted in metabolic cages (Oxymax; Columbus Instruments, USA), as previously described[4]. The chambers were equipped with two-dimensional infrared beam sensors (Opto-M3;

Columbus Instruments, USA) for locomotor activity measurements. Total energy expenditure (TEE) was calculated as oxygen consumption (VO_2) × (3.815 + 1.232 × RQ), where RQ is the respiratory quotient [the ratio CO_2 production (VCO_2)/VO_2]. Net fat oxidation rate was calculated using the formula by Simonson and DeFronzo[5]: Fat oxidation = 1.69 × (VO_2–VCO_2). Values were normalized with respect to the body weight and adjusted to an effective metabolic body size ($kg^{0.75}$).

Gene expression Analysis

Total RNA was isolated from 50 mg of frozen liver and 100 mg of frozen fat tissues using a Trizol® from Life Technologies (Grand Island, NY), according to the manufacturer's recommendations. The concentration of RNA samples was measured by Nanodrop® ND-1000 (Thermo Scientific, Wilmington, DE). Real-time quantitative PCR amplifications were carried out in 7900HT Sequence Detection System from Applied Biosystems (Foster City, CA) and Eco Real-Time PCR system from Illumina (San Diego, CA) in a 20 µl volume. The reaction was conducted using Power SYBR® Green RNA-to-CT™ 1-Step Kit from Life Technologies (Grand Island, NY) following the manufacturer's recommendations. Both of the forward and reverse primers (200 nM each), and 40 ng of template RNA were used. All reactions were carried out in four biological replicates. The PCR amplifications were conducted by the manufacturer's recommendations. To distinguish specific amplicons from non-specific amplifications, a dissociation curve was generated and examined. The Ct-values were calculated with SDS 2.3, RQ Manager 1.2 (Applied Biosystems), and Eco® software V4.0 (Illumina) with an automatic adjustment of base line and determination of Ct. The resulting Ct-values were imported to Microsoft Excel worksheet for further analysis. Statistical analysis was conducted using Graphpad

Prism software (GraphPad Software Inc.). The primers used for Collagen and TGF-β were designed by using Primer-BLAST software[6] (www.ncbi.nlm.nih.gov/tools/primer-blast/). The primer sequences were designed to span the intron region of a target gene to avoid amplification of trace amounts of genomic DNA in the samples. The sequences were as follows: collagen 1a1 (Col1a1)-F, CTGACGCATGGCCAAGAAGA, Col1a1-R, ATACCTCGGGTTTCCACGTC; TGF-β-F, ACGTCACTGGAGTTGTAGG, TGF-β-R, ATGTCATGGATGGTGCCCAG; β-actin-F, TTTGCAGCTCCTTCGTTGCC, and β-actin-R, ACGGTTGGCCTTAGGGTTCAG.

Reference

1. Abdelmegeed, M.A. *et al.* PPARalpha expression protects male mice from high fat-induced nonalcoholic fatty liver. *J Nutr* **141**, 603-610 (2011).
2. Abdelmegeed, M.A. *et al.* Critical role of cytochrome P450 2E1 (CYP2E1) in the development of high fat-induced non-alcoholic steatohepatitis. *J Hepatol* **57**, 860-866 (2012).
3. Abdelmegeed, M.A., Moon, K.H., Hardwick, J.P., Gonzalez, F.J. & Song, B.J. Role of peroxisome proliferator-activated receptor-alpha in fasting-mediated oxidative stress. *Free Radic Biol Med* **47**, 767-778 (2009).
4. Tam, J. *et al.* Peripheral CB1 cannabinoid receptor blockade improves cardiometabolic risk in mouse models of obesity. *J Clin Invest* **120**, 2953-2966 (2010).
5. Simonson, D.C. & DeFronzo, R.A. Indirect calorimetry: methodological and interpretative problems. *Am J Physiol* **258**, E399-412 (1990).
6. Ye, J. *et al.* Primer-BLAST: a tool to design target-specific primers for polymerase chain reaction. *BMC Bioinformatics* **13**, 134 (2012).

Supplementary Figures S1-6

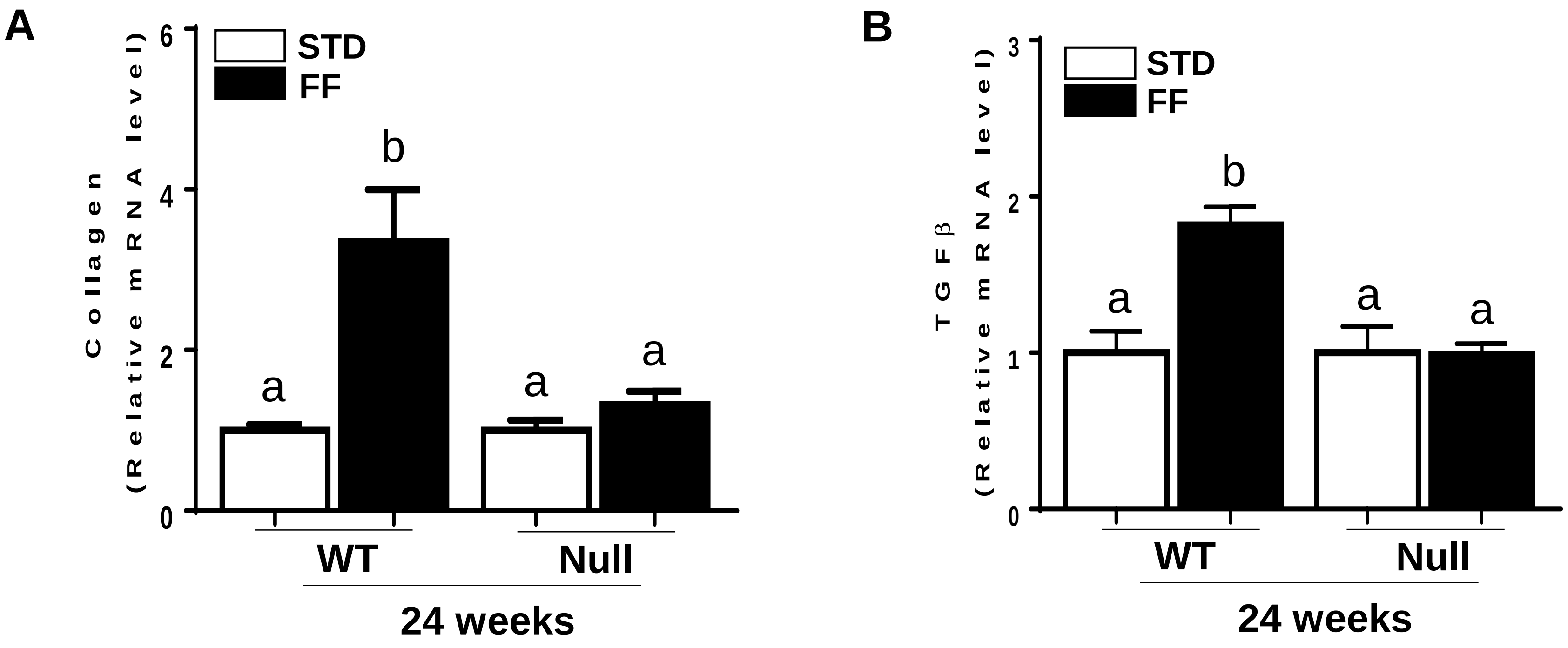

Figure S1. Increased mRNA levels of collagen and TGF-β in WT-FF at 24 wks.
Relative mRNA levels of the two genes involved in fibrosis: (A) collagen and (B) TGF-β are shown. Relative expression of each target mRNA has been standardized to β-actin mRNA. Data are presented as mean ± SEM (*n* = 4/group). Columns without a common small alphabetical character are significantly different from the other group(s).

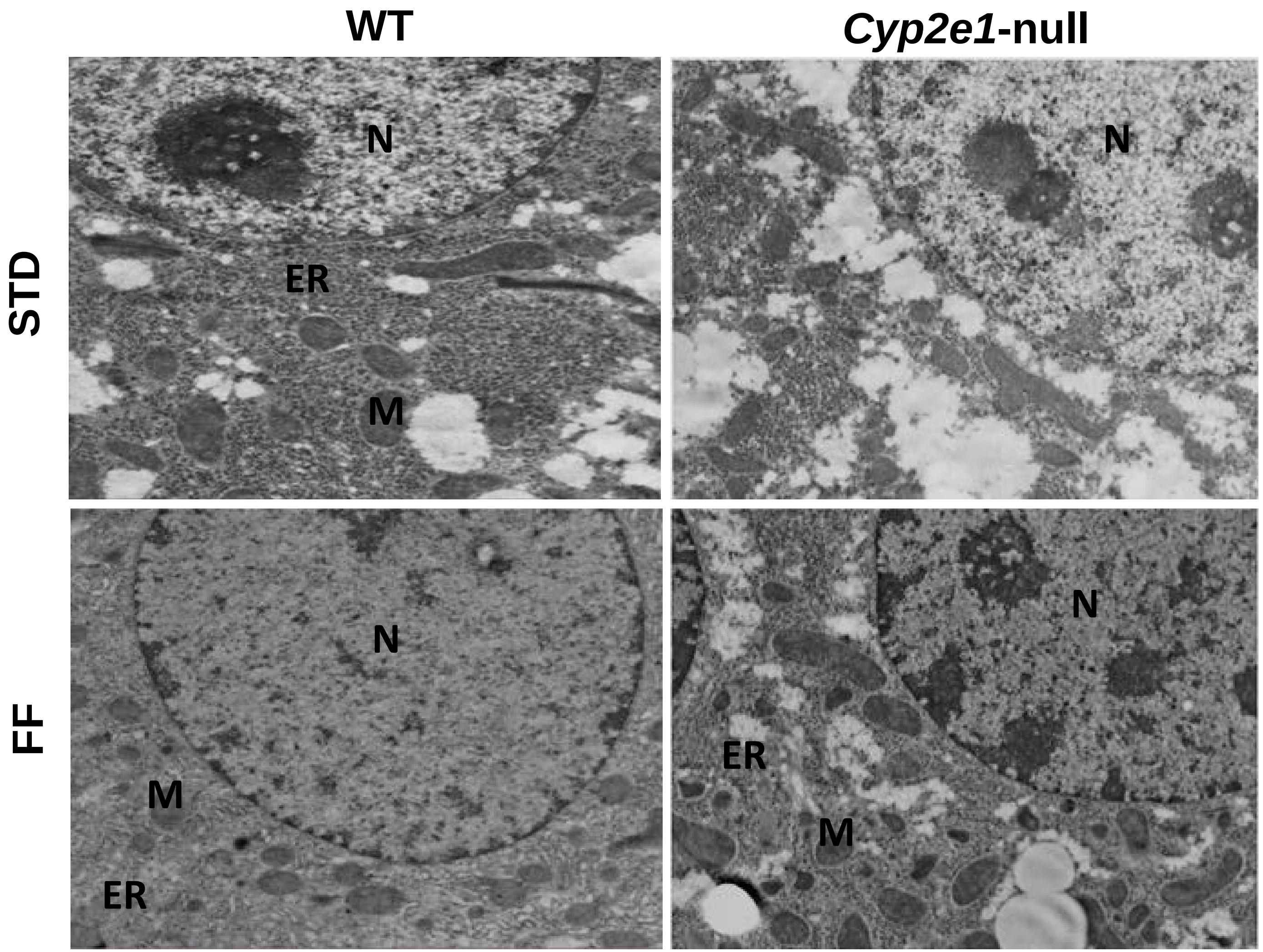

Figure S2. Ultrastructural features of FF-induced liver injury. Transmission EM studies demonstrating regular parallel organized ER (ER) in close association with mitochondria (M) in control (STD) livers in WT and *Cyp2e1*-null mice (N, nucleus) and irregularly arranged and disrupted ER, particularly in WT-FF. There was slight variability in mitochondrial size and shape in FF groups.

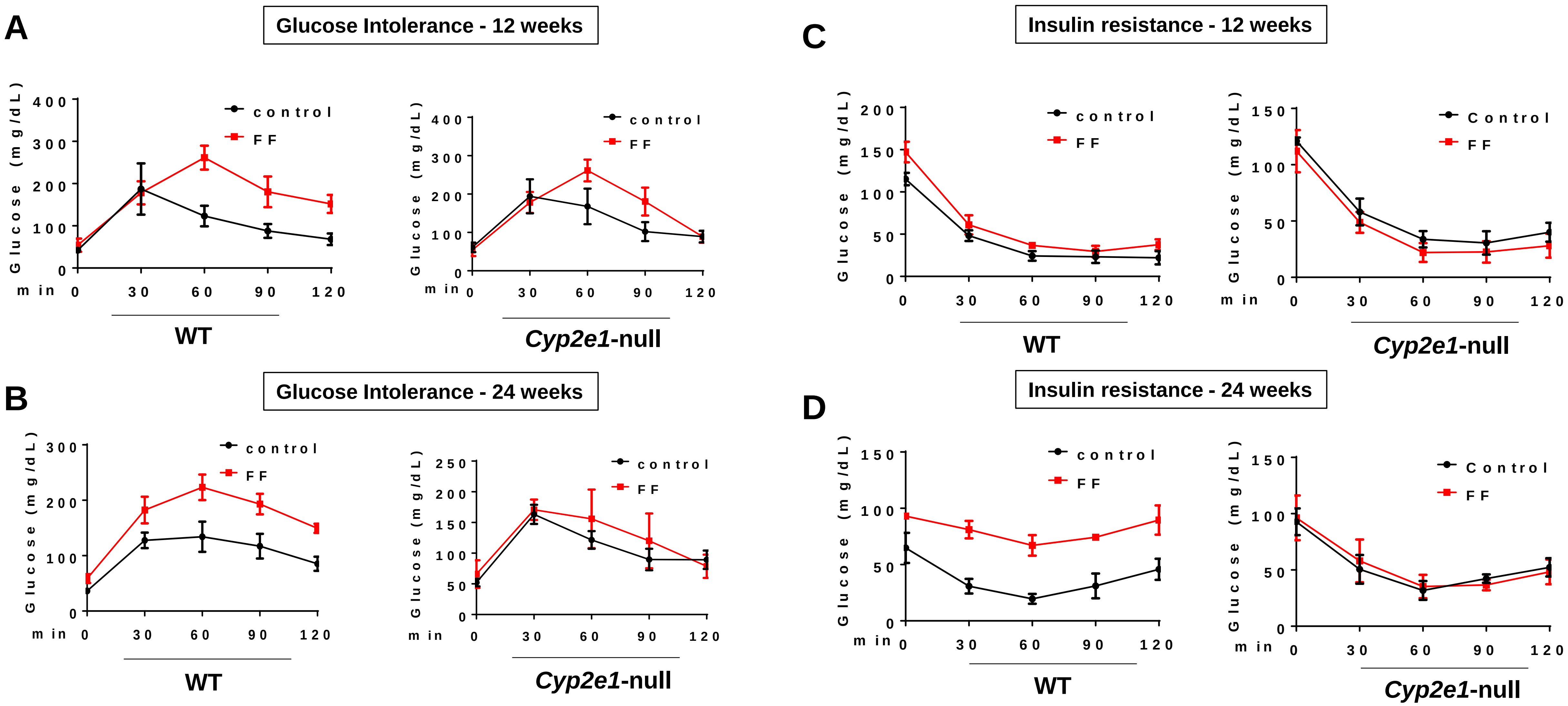

Figure S3. Impaired GT and increased IR in WT-FF.
Tail blood was collected following glucose injection (i.p. 2 g/kg) (A and B) or insulin (i.p. 0.75 U/kg) (C and D) at the indicated time points for all groups, as illustrated (*n*=4/group). Statistical differences for AUC are shown in Fig. 7

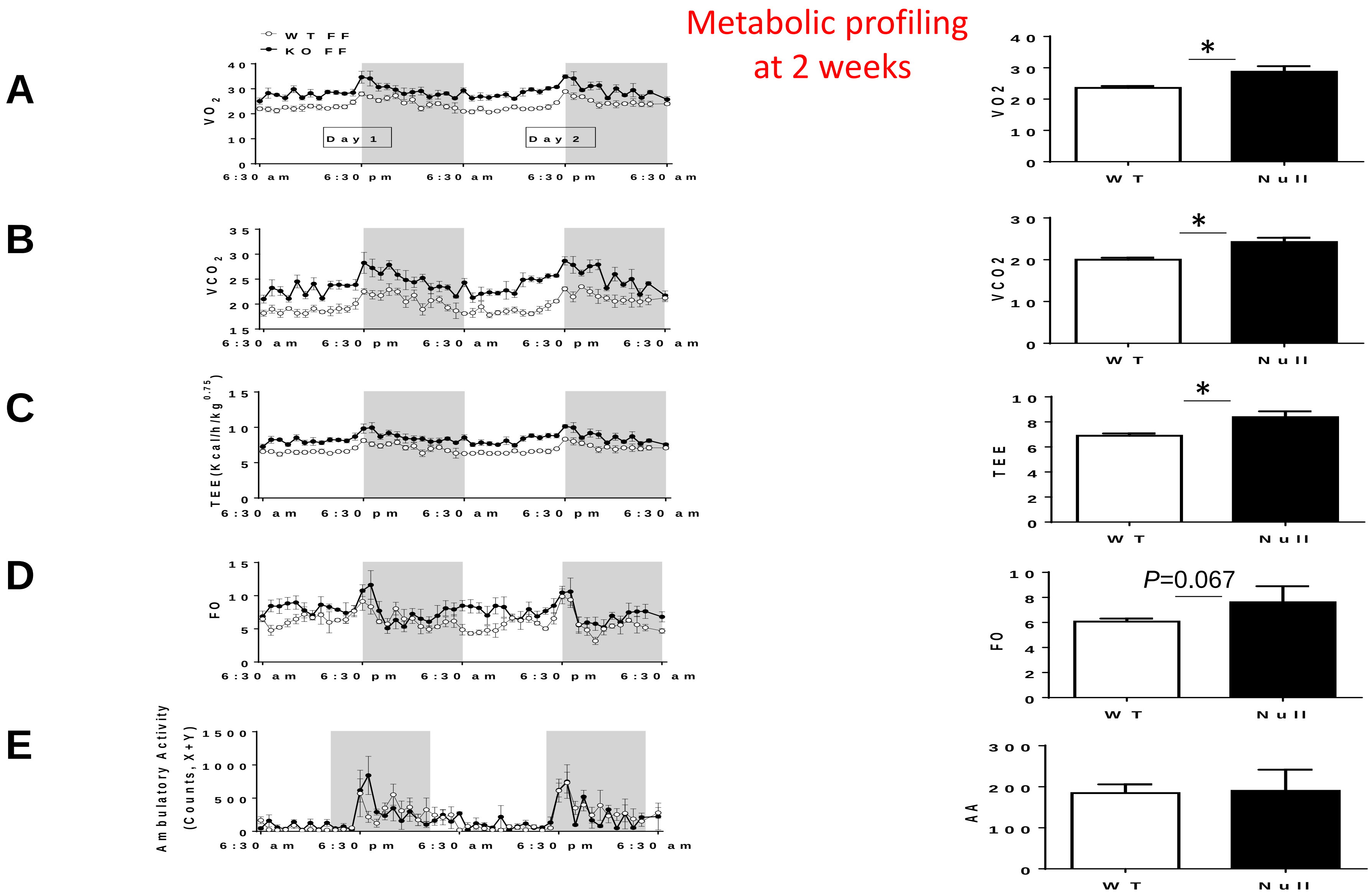

Figure S4. Metabolic profiles of FF-fed mice for 2 weeks as analyzed by means of indirect calorimetry.
Data were monitored by indirect calorimetry for WT-FF and null-FF and presented as hourly observations (right panels; shaded area indicates lights off) or as average of a 48-hours recording period (left panels). The rates of (A) O2 consumption, (B) CO2 production, (C) total energy expenditure (TEE), (D) fat oxidation (FO), and (E) ambulatory activity (AA) are presented. *n* = 3-4 samples/group, *P < 0.05.

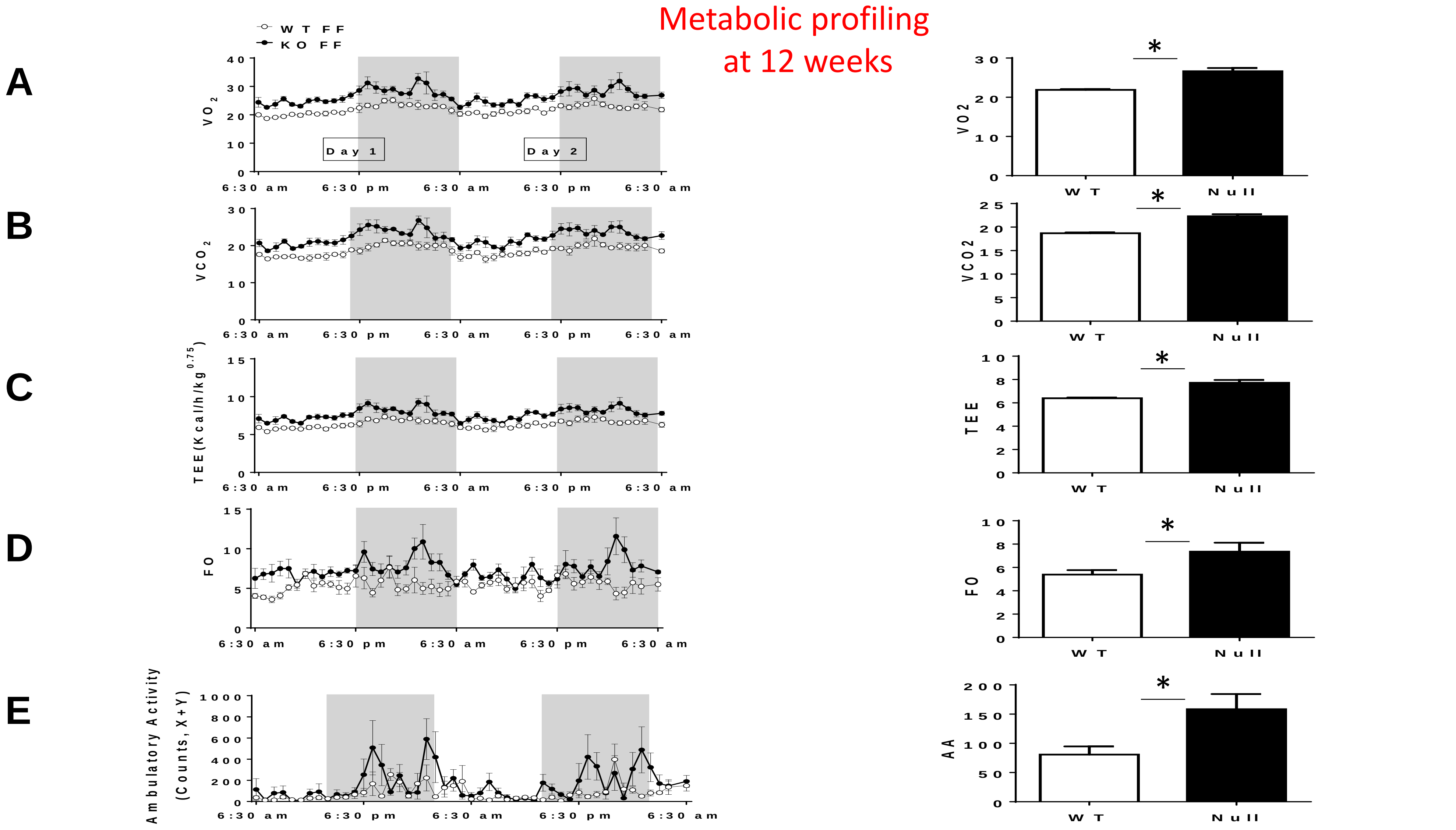

Figure S5. Metabolic profiles of FF-fed mice for 12 weeks as analyzed by means of indirect calorimetry.
Data were monitored by indirect calorimetry for WT-FF and null-FF and presented as hourly observations (right panels; shaded area indicates lights off) or as average of a 48-hours recording period (left panels). The rates of (A) O2 consumption, (B) CO2 production, (C) total energy expenditure (TEE), (D) fat oxidation (FO), and (E) ambulatory activity (AA) are presented. *n* = 3-4 samples/group, *P < 0.05.

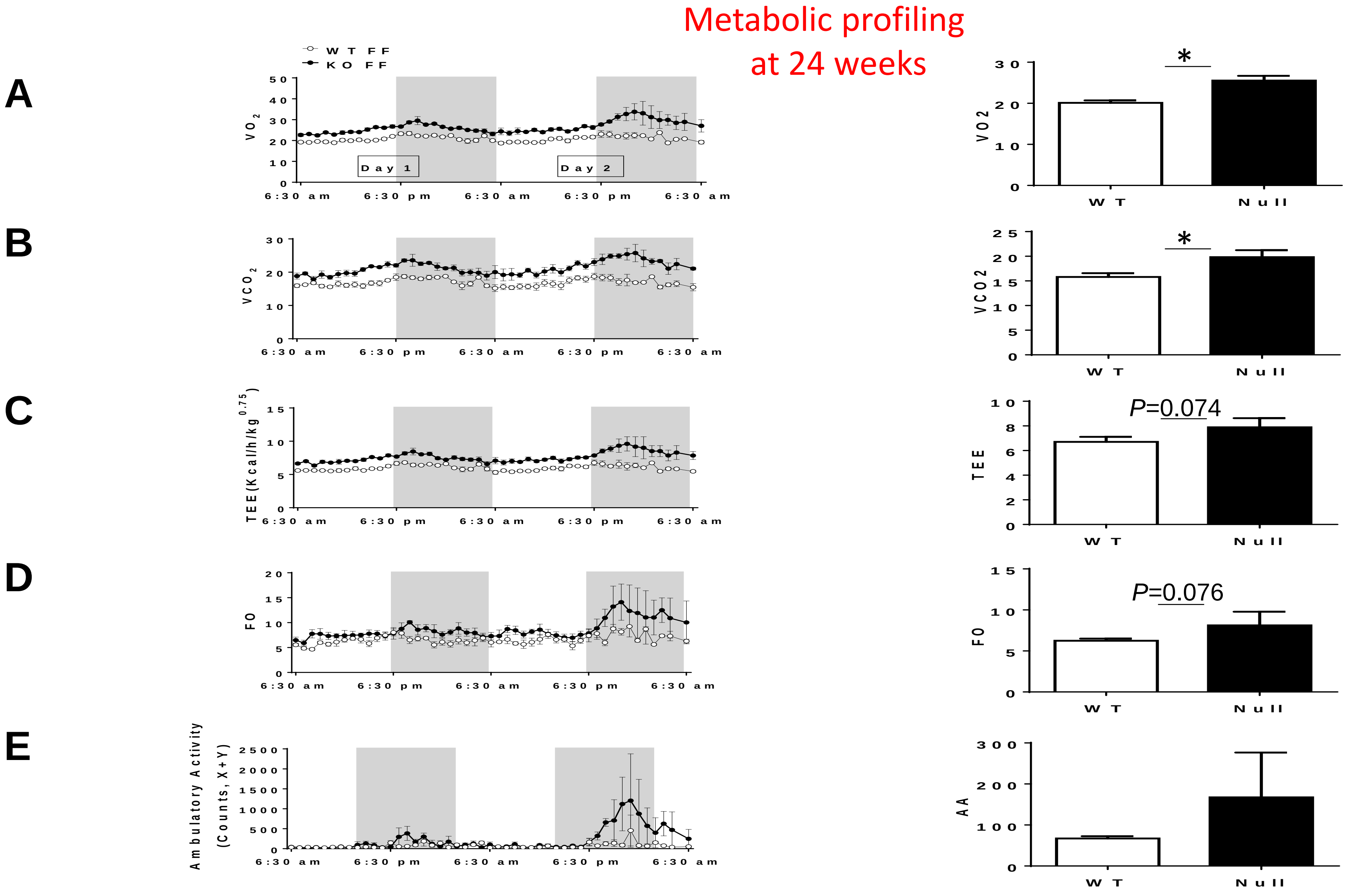

Figure S6. Metabolic profiles of FF-fed mice for 24 weeks as analyzed by means of indirect calorimetry.
Data were monitored by indirect calorimetry for WT-FF and null-FF and presented as hourly observations (right panels; shaded area indicates lights off) or as average of a 48-hours recording period (left panels). The rates of (A) O2 consumption, (B) CO2 production, (C) total energy expenditure (TEE), (D) fat oxidation (FO), and (E) ambulatory activity (AA) are presented. *n* = 3-4 samples/group, *P < 0.05.
